# Mixed-reality simulation for orthognathic surgery

**DOI:** 10.1186/s40902-016-0059-z

**Published:** 2016-03-09

**Authors:** Kenji Fushima, Masaru Kobayashi

**Affiliations:** 1 Division of Orthodontics, Department of Highly Advanced Stomatology, Graduate School of Dentistry, Kanagawa Dental University, 3-31-6 Tsuruya-cho, Kanagawa-ku, Yokohama, Kanagawa 221-0835 Japan; 2 Department of Oral & Maxillofacial Surgery, Graduate School of Dentistry, Kanagawa Dental University, 82 Inaoka-cho, Yokosuka, Kanagawa 238-8580 Japan

**Keywords:** Orthognathic surgery, Mixed-reality simulation, Computed tomography, Motion tracking, Facial asymmetry, Dental compensation

## Abstract

**Background:**

Mandibular motion tracking system (ManMoS) has been developed for orthognathic surgery. This article aimed to introduce the ManMoS and to examine the accuracy of this system.

**Methods:**

Skeletal and dental models are reconstructed in a virtual space from the DICOM data of three-dimensional computed tomography (3D-CT) recording and the STL data of 3D scanning, respectively. The ManMoS uniquely integrates the virtual dento-skeletal model with the real motion of the dental cast mounted on the simulator, using the reference splint. Positional change of the dental cast is tracked by using the 3D motion tracking equipment and reflects on the jaw position of the virtual model in real time, generating the mixed-reality surgical simulation. ManMoS was applied for two clinical cases having a facial asymmetry. In order to assess the accuracy of the ManMoS, the positional change of the lower dental arch was compared between the virtual and real models.

**Results:**

With the measurement data of the real lower dental cast as a reference, measurement error for the whole simulation system was less than 0.32 mm. In ManMoS, the skeletal and dental asymmetries were adequately diagnosed in three dimensions. Jaw repositioning was simulated with priority given to the skeletal correction rather than the occlusal correction. In two cases, facial asymmetry was successfully improved while a normal occlusal relationship was reconstructed. Positional change measured in the virtual model did not differ significantly from that in the real model.

**Conclusions:**

It was suggested that the accuracy of the ManMoS was good enough for a clinical use. This surgical simulation system appears to meet clinical demands well and is an important facilitator of communication between orthodontists and surgeons.

## Background

The purpose of orthognathic surgery is not only to improve jaw morphology but also to correct the inter-occlusal relationship. In patients with jaw deformities, therefore, three-dimensional diagnosis and simulation of skeletal and occlusal problems seem to be essentially important [1–4]. We have developed a new orthognathic surgical simulation system, named mandibular motion tracking system (ManMoS) [5, 6]. ManMoS uniquely integrates the real motion of the dental cast model with the virtual motion of the reconstructed craniofacial model, generating the so-called mixed-reality simulation. The skeletal change of the jaw osteotomy is simulated on the PC monitor while the occlusal change is confirmed by checking the cast model on the simulator. The simulation process is easily repeated, and the operator can make several attempts to determine the final jaw position.

Key points of the ManMoS are listed below.

- The importance of differential diagnosis of the dental compensation for jaw deformity in three dimensions
- Mixed-reality surgical simulation
- Jaw repositioning with the priority given to the skeletal correction rather than the occlusal correction
- Rigid internal fixation
- Surgery early

This article aimed to outline the ManMoS in two orthognathic surgery cases having a facial asymmetry and to examine the accuracy of the ManMoS.

## Methods

The ManMoS uniquely integrates the virtual dento-skeletal model with the real motion of the dental cast mounted on the simulator, generating the mixed-reality surgical simulation. The method to provide the surgical simulation system is described below. This research has been reviewed from an ethical respective by the ethics committee of the Kanagawa Dental University and approaved by the institute director (No.337).

### Reference splint

**Fig. 1:**
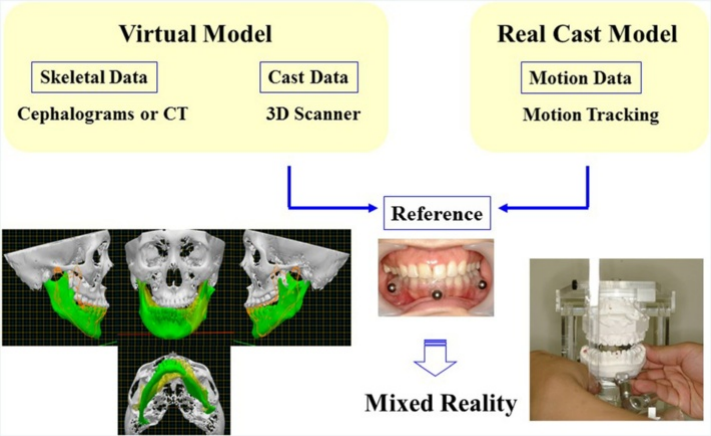
Mixed-reality surgical simulation system

**Fig. 2:**
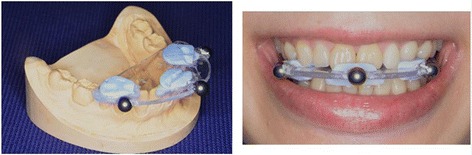
Reference splint

As explained in Fig. 1, ManMoS consists of the three-dimensional (3D) craniofacial model in virtual space and the real dental cast model. In order to integrate these different models, a reference splint is made by an acrylic plate and three titanium spheres are fixed with the plate approximately locating to the bilateral molar regions and incisal region (Fig. 2). The reference splint is adjusted so that a patient occludes it stably.

### Skeletal model

**Fig. 3:**
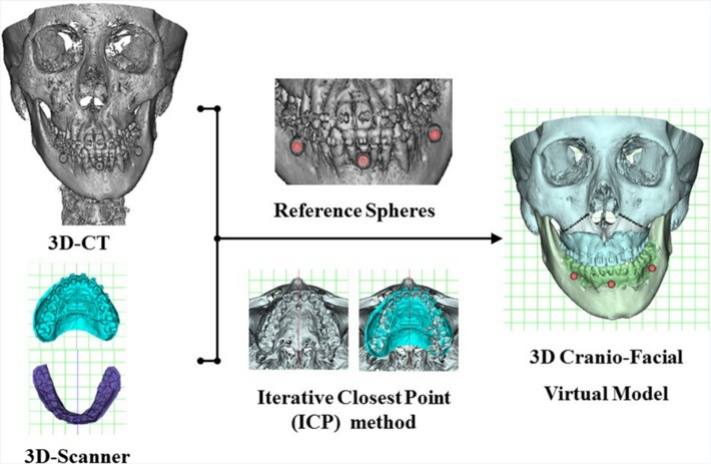
3D craniofacial model with reference spheres

Computed tomography (CT) is recorded while the patient occludes the reference splint. As shown in Fig. 3, 3D craniofacial model is reconstructed from the DICOM data of the CT recording. Three titanium spheres are automatically detected by the labeling method and available for the following integration process.

### Dento-skeletal model

**Fig. 4:**
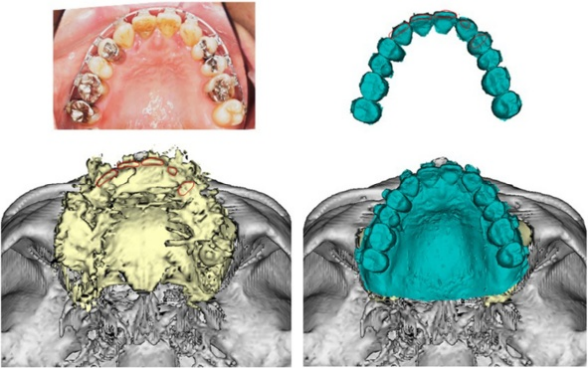
Dento-skeletal model

In 3D-CT model, the image of dentition is often disturbed due to a metal artifact. Figure 4 is an example in which the upper dentition is remarkably disturbed. In ManMoS, precise dental cast models are obtained by 3D scanner. On the CT image, areas without a metal artifact are found out. Corresponding areas on the 3D-scanned dental image are superimposed by using the iterative closest point algorithm. The dental area of 3D-scanned image replaces that of 3D-CT; consequently, dento-skeletal model with the reference spheres is reconstructed as a virtual model (Fig. 3).

### Motion capture of real dental cast model

**Fig. 5:**
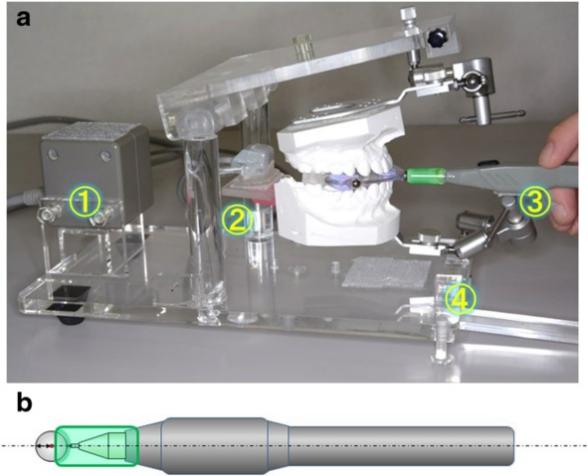
3D motion tracking and digitizing equipment. **a** ① Transmitter; ② box-type receiver; ③ stylus receiver; ④ universal joint. **b** Geometrical location of the stylus receiver to the titanium reference sphere

ManMoS uses the 3D motion tracking and digitizing equipment Fastrak (Polhemus Co. USA) (Fig. 5), consisting of a system electronics unit (SEU), a transmitter producing a magnetic field, and receivers to detect its own position. During recording, the SEU sends the dataset of three coordinates (X, Y, and Z) and three Oylar’s angles (azimuth, elevation, and roll) with a time resolution of 16.6 ms (60 Hz). Two types of receiver are prepared for this simulation. In order to track cast motion during surgical simulation, the box-type receiver is fixed on the dental casts and the transmitter is placed at the rear of the simulator. During the simulation, the receiver tracks the position of the real cast model in the magnetic field generated by the transmitter.

### Mixed-reality surgical simulation

At the beginning of the simulation, the upper and the lower dental casts are mounted on the simulator while the reference splint is intermaxillary placed between the casts. The stylus receiver is used to digitize three spheres on the reference splint (Fig. 5a), which are also detected in the virtual space of dento-skeletal model (Fig. 3). A bowl-shaped cover is worn on the tip of the stylus receiver, so that the tip of the stylus touch to the sphere’s surface and the long axis of the stylus orients to the center of the sphere (Fig. 5b). Since the diameter of the sphere is 6.0 mm, the center of the sphere locates at 3.0 mm ahead of the tip of the stylus along its long axis. As referred to center coordinates of these spheres, ManMoS integrates the motion data of the real cast model with the virtual dento-skeletal model; consequently, the mixed-reality simulation for an orthognathic surgery is set up (Fig. 1).

ManMoS was applied for two orthognathic surgery cases treated by the sagittal splitting ramus osteotomy (SSRO).

### Measurement errors

In order to assess the accuracy of ManMoS, positional change of the lower dental arch during the simulation was compared between the virtual and the real model.

At the initial mandibular posture, three landmark points of the central incisor and the bilateral first molars were measured on the lower dental arch of the virtual model. Corresponding points on the real dental cast were also measured directly by using the stylus receiver of the Fastrak. Then, the lower dental cast was arbitrarily moved to the simulator, and three landmark points at the simulated posture were measured both on the real cast and on the virtual lower dental arch in ManMoS. Linear moving distance of each landmark points from the initial to the simulated mandibular posture was calculated and compared between the virtual and the real measurements.

This process was repeated 25 times. Wilcoxon duplicate determination was performed, and the errors of measurement were established.

## Results

### Measurement error

Table 1 shows the linear moving distance of each landmark points in ManMoS simulation. Their linear moving distance measured on the virtual dental model in ManMoS was not significantly different from those measured on the real dental cast. With the measurement data of the real lower dental cast as a reference, measurement error of the incisal, the right molar, and the left molar on the virtual model was 0.32, 0.31, and 0.27 mm, respectively.

**Table 1:** Linear moving distances of measurement points (mm)

	Accuracy of ManMoS simulation								
*n* = 25	ManMoS			FASTRAK			Difference between ManMoS and FASTRAK (absolute value)		
	Incisor	Right molar	Left molar	Incisor	Right molar	Left molar	Incisor	R_M	L_M
Average	9.43 n.s.	8.99 n.s.	9.44 n.s.	9.50	8.89	9.58	0.39	0.37	0.31
Standard deviation	3.41	3.30	3.18	3.45	3.47	3.17	0.24	0.24	0.22
Minimum	3.71	0.79	2.54	3.38	0.19	2.71	0.04	0.00	0.01
Maximum	14.51	13.85	15.49	15.22	13.65	15.56	0.75	0.83	0.77

*n.s.* no significant difference at 5 % level (Wilcoxon signed-rank test)

### Case 1

#### Facial and oral findings

**Fig. 6:**
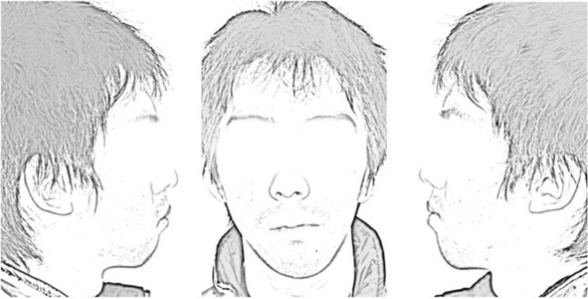
Facial photographs (initial record) in case 1

**Fig. 7:**
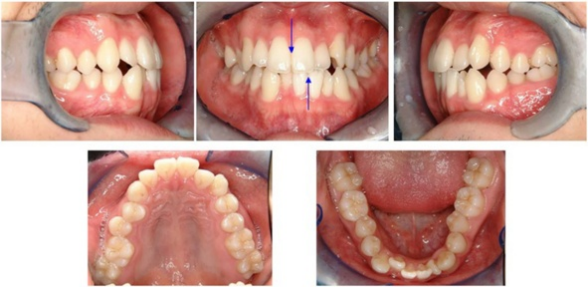
Oral photographs (initial record) in case 1

ManMoS was applied to a facial asymmetry case. In the frontal view of facial photographs (Fig. 6), facial asymmetry appeared to show chin deviation toward the left and cant of the lip ascended toward the left. In the lateral facial photographs, dolichofacial pattern with large lower facial height is found. Oral photographs show dental asymmetries (Fig. 7). The lower denture midline is deviated from the upper one to the left. For the Angle’s molar relationship, class III relationship is found on the right while class I on the left. The upper dental arch is constricted showing V-shaped arch.

#### Coordinate system for 3D diagnosis

**Fig. 8:**
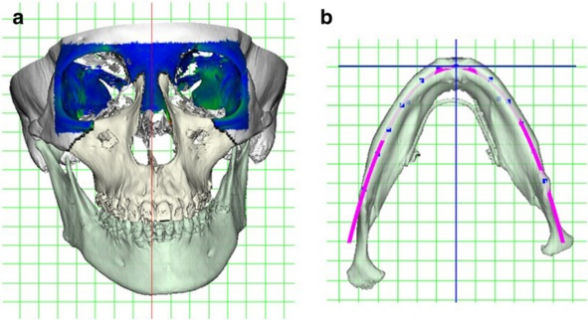
Reference coordinate system for 3D diagnosis. **a** Cranial global coordinates. **b** Mandibular local coordinates MLB

Prior to the surgical simulation, three-dimensional diagnosis of the skeletal and dental problems was performed. Especially for facial asymmetry cases, to set up global and local reference coordinates is fundamentally important to assess the skeletal and dental asymmetries in three dimensions. Cranial global coordinates and mandibular local coordinates are available in ManMoS. For the cranial coordinates, ManMoS refers to the Orbita area using a mirroring technique, in which the mid-sagittal plane is determined so that the difference between the right and left Orbita is smallest (Fig. 8a). For the mandibular coordinates, a best fitting arch of a quartic curve to the mandibular lower border is set up and abbreviated as MLB (Fig. 8b).

#### Skeletal problems

**Fig. 9:**
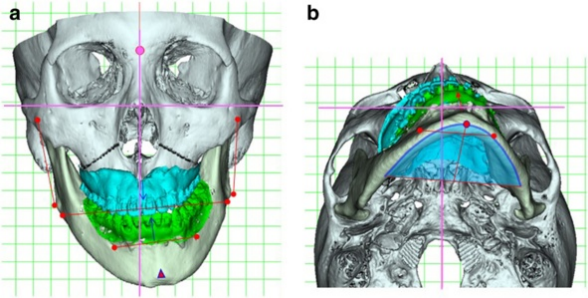
3D diagnosis in cranial coordinates in case 1. **a** Frontal view. **b** Inferior view

**Fig. 10:**
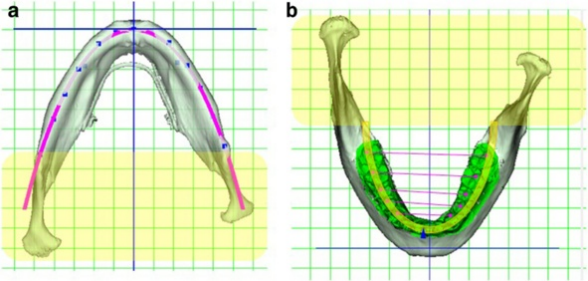
3D diagnosis in mandibular local coordinates MLB in case 1. **a** Inferior view. **b** Superior view

Figure 9 demonstrates the skeletal problems in the cranial coordinates, which are the mandibular lateral displacement toward the left accompanied with rotation both in the frontal (Fig. 9a) and the inferior view (Fig. 9b). When the mandibular asymmetry is assessed in the local coordinates of MLB (Fig. 10), remarkable asymmetry is not found at the tooth-bearing areas of the mandible, but found at the ramus areas.

#### Dental problems

For the dental problems assessed in the cranial coordinates (Fig. 9), the lower denture midline is deviated to the left and mesial occlusal relationship on the right side is found along with the mandibular displacement.

**Fig. 11:**
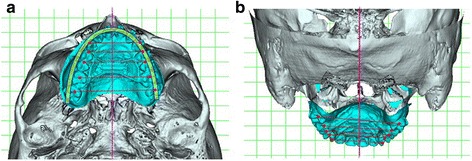
Upper dental or maxillary compensation for mandibular asymmetry. **a** Inferior view. **b** Posterior view

For the dental problems of the maxilla (Fig. 11), the upper arch width on the right side is constricted compared with that on the left. Occlusal plane in the posterior view is ascended toward the left.

Asymmetries in the lower dental arch are assessed in the local MLB coordinates (Fig. 10). For the surgical simulation in ManMoS, in order to correct the facial asymmetry, the MLB (tooth-bearing area of the mandible) should be repositioned symmetrically against the cranial global coordinates. The lower dental arch should be symmetrically aligned referring to the MLB coordinates. As shown in Fig. 10b, the lower denture midline is displaced 3.0 mm toward the right. As referred to symmetrical arch, the left lateral teeth are positioned more mesially and lingually than those in the opposite side.

#### Mixed-reality simulation for orthognathic surgery

Following the measurements of the three reference spheres, mixed-reality simulation was started. When the operator handles the real cast model, the virtual model of the mandible or the maxilla follows the corresponding dental cast movement in real time. The skeletal change is simulated on the PC monitor, while the occlusal change is confirmed by checking the cast model on the simulator (Fig. 1).

**Fig. 12:**
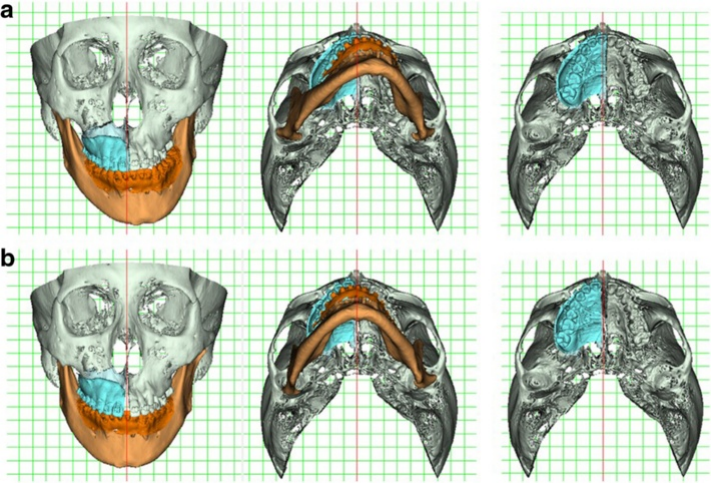
3D simulation in case 1. **a** Original model. **b** Simulated model. Mandible: SSRO. Maxilla: unilateral RME assisted by Le Fort I corticotomy

In this case, mandibular asymmetry was corrected by SSRO. As shown in Fig. 12, tooth-bearing segment of the mandible was simulated to be placed symmetrically in the cranial coordinates. As a result of the mandibular simulation, posterior crossbite appeared on the right side, indicating constriction of the upper arch width at the right side. Therefore, for the maxilla, Le Fort I corticotomy was applied on the right side and unilateral rapid maxillary expansion (RME) was performed.

#### Treatment progress

**Fig. 13:**
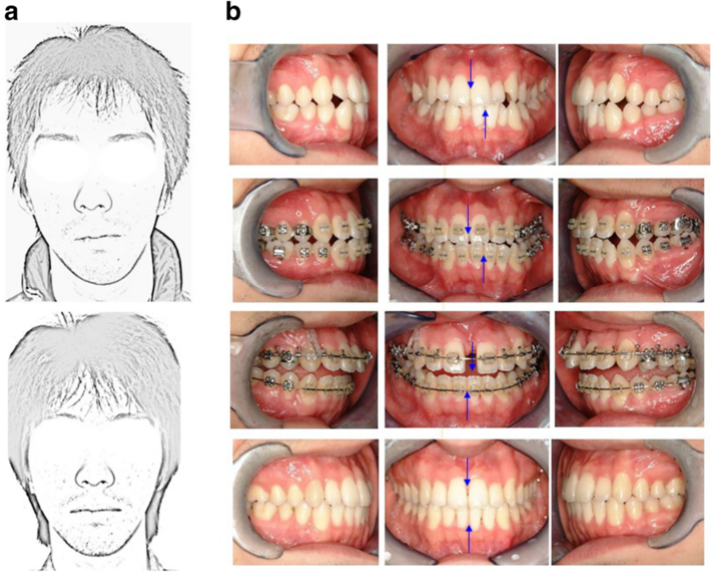
Treatment progress in case 1. **a** Facial photographs of pre- and post-treatment. **b** Oral photographs at initial, 6 months (pre-surgery), 7 months (2 weeks after surgery), and 1 year 1 month (post-treatment)

Figure 13 shows the treatment progress in the facial and oral photographs. The basic concept of the ManMoS is “surgery early”; in this case, it took only 6 months for the active orthodontic treatment before surgery. As shown in the oral photographs 2 weeks after surgery, occlusal condition seems to be unstable due to the ManMoS simulation giving the priority to the skeletal correction rather than the occlusal correction. The unstable occlusal condition was resolved in a few months, and orthodontic treatment was completed 7 months after surgery.

### Case 2

ManMoS occasionally makes us notice the application of unilateral mandibular osteotomy.

#### Facial and oral findings

**Fig. 14:**
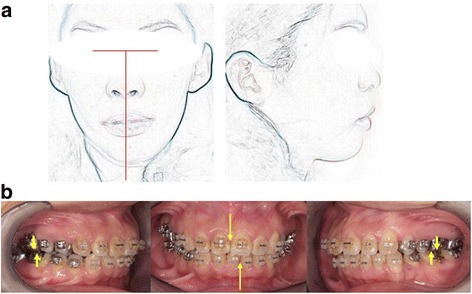
Photographs (just before surgery) in case 2. **a** Facial photographs. **b** Oral photographs

Figure 14 demonstrates facial and occlusal findings just before surgery. Chin deviation toward the left with the cant of the lip line is found in the frontal facial photograph. The occlusal photographs show that the lower denture midline is deviated toward the left.

#### 3D diagnosis of skeletal problems

**Fig. 15:**
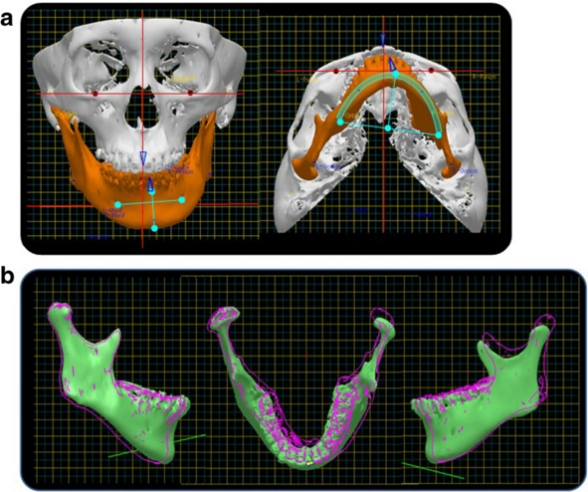
3D diagnosis and simulation in case 2. **a** Original model. **b** Superimposition of the simulated model on the original one (*solid line*). ManMoS demonstrates that the skeletal midline of the mandible is moved toward the right while the left half of the mandible is moved antero-inferiorly. Note that the right condylar position is not changed so much

3D-CT model was diagnosed in the cranial coordinate system. As the skeletal problems shown in Fig. 15a, the mandibular skeletal midline is displaced to the left accompanied with the rotation in frontal and inferior view.

#### Unilateral SSRO

**Fig. 16:**
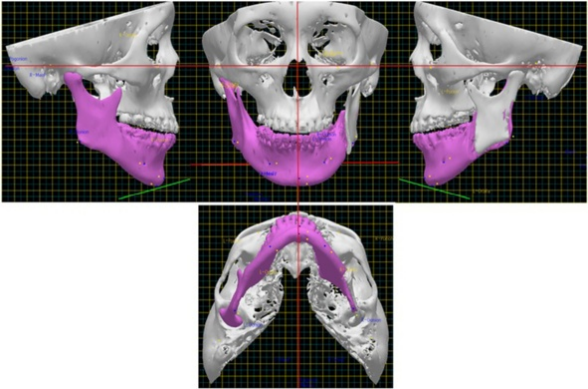
Unilateral SSRO

Mixed-reality simulation was carried out to correct the mandibular asymmetry. When the simulated mandibular position was compared to the original one, it was noticed that right condyle was not displaced so much (Fig. 15b). Simulation by ManMoS had made us a decision to apply the SSRO unilaterally only to the left side (Fig. 16). It is demonstrated that mandibular asymmetry is corrected well in the frontal and the inferior view. The tooth-bearing segment on the left is advanced antero-inferiorly around 7.0 mm.

#### Virtual cephalometric image

**Fig. 17:**
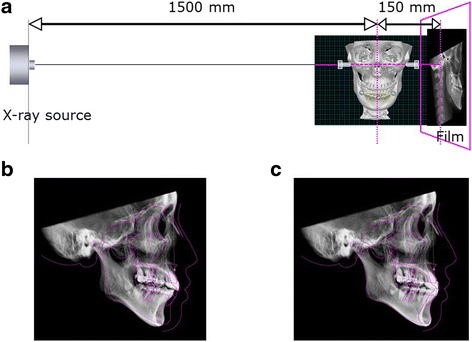
Virtual cephalometric image (VCI). **a** VCI reconstructed from 3D craniofacial model. **b** VCI of original model. **c** VCI of simulated model

For the assessment in the lateral view, virtual cephalometric image (VCI) is available in ManMoS. Two-dimensional VCI is reconstructed from 3D-CT craniofacial model. Lateral cephalometric projection is virtually simulated reconstructing the geometrical condition of the X-ray source, the craniofacial object, the ear rod, and the film (Fig. 17a). VCI reconstructed from 3D-CT model of the original and the final mandibular position were evaluated by means of the craniofacial drawing standard (Fig. 17b and c). It was confirmed that the mandibular repositioning on the lateral view was properly simulated.

#### Treatment progress

**Fig. 18:**
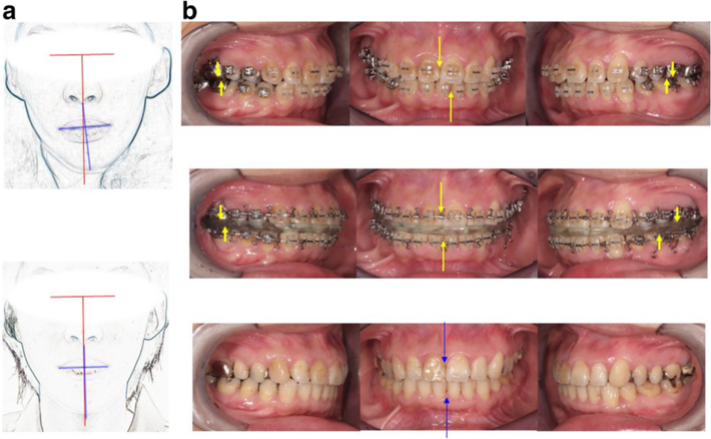
Treatment progress in case 2. **a** Facial photographs of pre and post treatment. **b** Oral photographs at initial, post-surgery, and post-treatment

Treatment progress are found in facial Photographs (Fig. 18a) and oral photographs of pre-surgery, post-surgery, and after an active orthodontic treatment (Fig. 18b). It is found that the application of unilateral SSRO had successfully improved facial asymmetry and malocclusion.

## Discussion

### Measurement error

Since the mixed-reality surgical simulation system ManMoS comes into being through a series of complex operation, there are several error factors. It was confirmed that the accuracy and precision of the tracking device Fastrak itself appeared to be good enough [5]. In the linear measurements of 100 mm, the average error between the measured data and the actual value ranged from −0.308 to 0.136 mm. In this study, with the measurement data of the real lower dental cast as a reference, measurement error for the whole simulation system was less than 0.32 mm. It was suggested that the accuracy of the whole system was sufficient for clinical demands.

### Ramus asymmetry

As shown in case 1, chief characteristic of a facial asymmetry is the lateral displacement of the mandibular midline. Diagnosis in MLB reference (Fig. 10) represented that remarkable asymmetry was not found at the tooth-bearing part of the mandible, but found at the ramus parts. Therefore, facial asymmetry seems to be mandibular asymmetry, and mandibular asymmetry seems to be ramus asymmetry. And in most cases having a facial asymmetry, the tooth-bearing part of the mandible considers to be displaced due to the asymmetrical growth of the ramus.

### Dental compensation

It was reported that characteristic dental asymmetries were found in patients with a facial asymmetry [3,4]. As dental asymmetry shown in case 1, the lateral deviation of the lower denture midline and the right-left difference of Angle’s molar relationship were due to the skeletal asymmetry (Fig. 9). Therefore, they should be corrected by means of a jaw surgery. When the lower dental arch was assessed in the mandibular local reference MLB (Fig. 10), the left lateral teeth was inclined lingually. This finding is considered to be a dental compensation commonly found in the side of chin deviation and should be corrected by means of orthodontic treatment. ManMoS provides the differential diagnosis whether dental asymmetries should be corrected by means of a surgery or an orthodontics clearly.

**Fig. 19:**
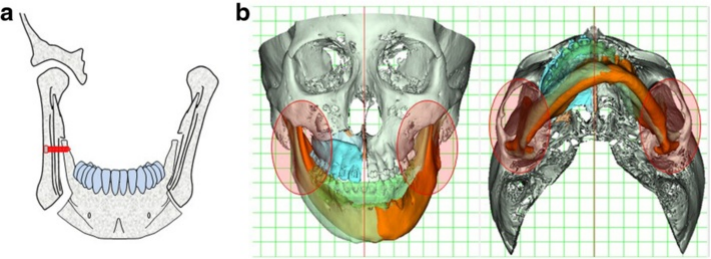
Ramus positioning. **a** Flaring out of the condylar proximal segment. **b** Superimposition of the simulated model on the original one

In ManMoS, the mandibular repositioning is simulated with priority given to the skeletal correction rather than the occlusal correction. With regard to SSRO, mediolateral displacement of the posterior portion of the distal tooth-bearing segment impinges on the condylar proximal segment, resulting in a large osteotomy gap between them (Fig. 19a) [7]. ManMoS allows for the original and simulated positions of the ramus to be compared in both the frontal and horizontal planes (Fig. 19b,c).

Accurate positioning of the ramus is essentially important and clearly prevents many of the problems associated with the impingement on the condylar proximal segment [8].

### Rigid internal fixation

In ManMoS, jaw repositioning is simulated based on the skeletal correction. Consequently, little occlusal contact occurs and the occlusion is unstable at the final mandibular posture (Fig.13b). Such unstable occlusal condition is a risk to produce oral discomfort and the relapse of the bony segments. To ensure the skeletal correction by the osteotomy, the rigid internal fixation of the bony segments is essentially proposed in ManMoS and usually performed bicortically with the titanium screws.

### Occlusal wafer splint

After deciding the final jaw position in ManMoS, the occlusal relationship is transferred with a bite registration material on the simulator and occlusal wafer splint for inter-maxillary fixation is easily fabricated. This seems to be another advantage to use the real cast models in ManMoS.

## Conclusions

ManMoS was introduced as mixed-reality simulation for orthognathic surgery.

The skeletal change of the jaw osteotomy is simulated on the PC monitor while the occlusal change is confirmed by checking the cast model on the simulator.

It was suggested that the accuracy of the whole simulation system was sufficient for clinical demands.

ManMoS is effective for the simulation of an orthognathic surgery, especially in cases having complicated skeletal and dental asymmetries.

ManMoS appears to meet clinical demands well and is an important facilitator of communication between orthodontists and surgeons.

## Consent

Written informed consent was obtained from the patients for the publication of this paper and any accompanying images.

## Abbreviations

3D: three-dimensional
CT: computed tomography
ManMoS: mandibular motion tracking system
MLB: quartic curve to the mandibular lower border
RME: rapid maxillary expansion
SEU: system electronics unit
SSRO: sagittal splitting ramus osteotomy
VCI: virtual cephalometric image
